# Role of microbial life history strategy in shaping the characteristics and evolution of host-microbiota interactions

**DOI:** 10.1093/ismejo/wraf168

**Published:** 2025-08-05

**Authors:** Nancy Obeng, Johannes Zimmermann, Anna Czerwinski, Janina Fuß, Hinrich Schulenburg

**Affiliations:** Department of Evolutionary Ecology and Genetics, University of Kiel, Kiel 24118, Schleswig-Holstein, Germany; Roche Pharma Research and Early Development, Infectious Disease, Roche Innovation Center Basel, F. Hoffmann-La Roche, Basel, Basel-Stadt, Switzerland; Department of Evolutionary Ecology and Genetics, University of Kiel, Kiel 24118, Schleswig-Holstein, Germany; Max Planck Institute for Evolutionary Biology, Plön 24306, Schleswig-Holstein, Germany; Cluster of Excellence Balance of the Microverse, Friedrich Schiller University, Jena 07745, Thüringen, Germany; Department of Evolutionary Ecology and Genetics, University of Kiel, Kiel 24118, Schleswig-Holstein, Germany; Institute of Clinical Molecular Biology, University of Kiel, Kiel 24118, Schleswig-Holstein, Germany; Department of Evolutionary Ecology and Genetics, University of Kiel, Kiel 24118, Schleswig-Holstein, Germany; Max Planck Institute for Evolutionary Biology, Plön 24306, Schleswig-Holstein, Germany

**Keywords:** host-microbiota interactions, microbial life history, metaorganism, Caenorhabditis elegans, Pseudomonas lurida, Ochrobactrum vermis

## Abstract

Many host-associated microbes are transmitted between individual hosts via the environment and, therefore, need to succeed both within a host and a connected environmental habitat. These microbes might invest differentially into the two habitats, potentially leading to fitness trade-offs and distinct life history strategies that ultimately shape the host-associated microbial communities. In this study, we investigated how the presence of distinct bacterial life history strategies affects microbiota characteristics along a host-associated life cycle, using the nematode host *Caenorhabditis elegans* and two naturally associated bacteria, *Pseudomonas lurida* and *Ochrobactrum vermis*, as an experimentally tractable model. Based on genomic life history prediction and experimental fitness characterizations, we identified distinct ecological strategies for the bacteria: whereas *P. lurida* dominated the free-living environment, *O. vermis* was more abundant in the host. Using mathematical modelling, experimental evolution, and whole genome sequencing, we next assessed whether the two distinct ecological strategies influence further adaptation to the host-associated life cycle. We found that (i) the host-specialist *O. vermis* did not further adapt to the two habitats, whereas (ii) the initially better environmental competitor *P. lurida* adapted to the life cycle, leading to its increased abundance in both environment and host. Evolutionary adaptation of *P. lurida* caused a shift in microbiota composition in the host, which in turn, resulted in a significant increase in host fitness. Overall, our results highlight the role of microbial life history strategies in shaping the characteristics and evolution of host–microbe interactions and suggest a potential selective advantage of better environmental competitors.

## Introduction

Animals are known to acquire microbial symbionts from the environment. In host association, these microbes can provide nutrients, influence host physiology, development, and reproduction, and thereby shape organismal fitness [1, 2]. Host association implies that microbes are capable of colonizing the host, persisting within it, and eventually being transmitted to the next host generation. For successful transmission, they additionally need to survive or proliferate in the environment. Such a biphasic life cycle is widespread among host-associated microbes and thus a common feature of host-associated microbiota [3]. In such a biphasic life cycle, microbes are challenged with selection by two distinct compartments, host and a connected environment [3]. Although considerable attention has focused on microbial strategies of host-association [3–5], it remains unclear how adaptation to the two habitats, external environment and host, are related.

Community evolution is fundamentally shaped by selection and diversification of its members. Although selection acts on deterministic fitness differences between individuals of a particular taxon, communities diversify as new variants emerge [6–8]. These processes also occur in microbial communities, where short-term ecological and long-term evolutionary dynamics often overlap [7, 9], possibly within both the host and the free-living environment [3]. In the short term, species sorting drives microbiota composition as bacteria specializing on either habitat may face costs in the other. In the long term, adaptive change of individual lineages can additionally influence microbiota composition. To date, relatively little is known about the relative impact of the two connected habitats on microbiota assembly and evolution. Recent theoretical work suggests that a single species evolving in a biphasic life cycle should either (i) invest in growth in the external environment or (ii) decrease migration from host (i.e. stay in the host) to optimize the microbe’s fitness [10]. Additional life-history strategies may be important in such host-associated life cycles, including as examples high competitiveness to enhance survival in either external and/or host-associated microbial communities, high stress tolerance to cope with any host responses (e.g. host immunity), or formation of some kind of dauer stage to similarly cope with host responses or absence of hosts in the external environment. To date, it is unclear how different strategies of co-existing microbes would affect an evolving community. The complexity of most microbiotas makes it difficult to infer the relative influence of host and environmental selection empirically, as biotic interactions between microbiota members cannot be linearly predicted with increasing complexity [11], yet they do influence each other’s fitness.

To address these current knowledge gaps, our study aimed at advancing our understanding of how distinct microbial life histories determine microbiota characteristics along a biphasic host-associated life cycle on both ecological and evolutionary timescales. We utilized a simple, yet experimentally tractable two-member microbiota model, consisting of a well-characterized pair of bacteria from the natural microbiota of the nematode host *Caenorhabditis elegans* [12–14]: *Pseudomonas lurida* MYb11, which provides immune-protection against pathogens [12, 15–18], and *Ochrobactrum vermis* MYb71, which efficiently colonizes the nematode [12, 17, 19–21]. These two bacteria were originally co-isolated from the natural worm strain *C. elegans* MY316 [12], they are representative members of the native microbiota of *C. elegans* [12, 17, 18, 22], they co-exist within worms and on agar [12–14], they or related taxa can be predominant components of *C. elegans* microbiota communities depending on environment and host genotype [17, 21, 23–26], and MYb11 was previously found to be an informative model to study the genetics of host adaptation [27]. In the current study, we first predicted life history strategies of the two microbes using genome-based inference. We then experimentally quantified relative bacterial fitness along a biphasic life cycle, in the host and free-living on agar, using mono- and co-culture experiments. Together this revealed microbiota member-specific life history trade-offs and resulting distinct life history strategies. We subsequently tested whether distinct life history strategies influence further adaptation to a host-associated life cycle, using a mathematical model, experimental evolution, and whole genome sequencing. Finally, we evaluated how any adaptive changes in the two-member microbiota affect host fitness.

## Materials and methods

### Bacterial and worm strains

In this study, we studied a two-member microbiota community *P. lurida* MYb11 and *Ochrobactrum vermis* MYb71, two key members of the natural *C. elegans* microbiota [12–14]. These species have been co-isolated from the natural *C. elegans* strain MY316 [12]. Both bacteria can colonize worms and be maintained on nematode growth medium agar (NGM) [28], and we could thus assay relative fitness (colony forming units/worm or CFU/agar plate) within hosts and the environment. For all experiments with ancestral bacterial isolates, we inoculated tryptic soy broth overnight cultures (28°C, 150 rpm) with single bacterial colonies. We subsequently washed cells in M9 buffer and adjusted optical densities (OD_600nm_) according to assay requirements. For competition experiments, bacterial species were co-inoculated at equal ODs. Relative species abundances were later determined via selective plating (on tryptic soy agar with 10 μg/ml kanamycin), as only MYb71 is kanamycin-resistant.

The host in this study was the natural *C. elegans* isolate MY316, recovered from a rotting apple in the Kiel Botanical Gardens [12]. For all experiments, we thawed worms from frozen stocks and performed colonization experiments with sterile and synchronized worms. In preparation, *C. elegans* was maintained on the non-colonizing food bacterium *Escherichia coli* OP50 according to standard protocols [28].

### Bacterial growth

Bacterial fitness in the NGM agar environment was measured as growth in terms of colony forming units (CFUs), as before [29]. For this, we inoculated 6 cm NGM plates with 50 μl bacterial suspension at OD = 0.1 and incubated plates at 20°C. CFUs were sampled at 24 and 72 hours. Growth data of MYb11 mono-cultures were previously published [29].

### Bacterial colonization of worms

Bacterial fitness in the host was measured as CFU per worm. Briefly, five synchronized L4 stage *C. elegans* were placed on a lawn of MYb11, MYb71, or both (6 cm NGM agar, 125 μl at OD_600_ = 2). After 3.5 days (20°C), worms were paralyzed and collected in M9 buffer with 0.025% Triton-100 and 25 mM antihelminthic tetramisole. After washing on a custom-made filter tip washing system [30], worms were collected in M9 buffer with Triton-100 and worm-free supernatant collected as background. Samples were then homogenized via bead beating, and CFUs quantified via serial dilution and plating on tryptic soy agar. CFU/worm were determined by subtracting background CFU from worm samples and dividing the total CFUs by the number of worms sampled. Colonization data of MYb11 mono-cultures were previously published [29].

### Early colonization, established colonization, persistence, and release in worms

Early colonization, established colonization, short-term persistence, and release from the worm were measured as CFUs detected in or released from *C. elegans* (see also [27]). For early colonization, we placed L4 worms previously raised on *E. coli* OP50 on bacterial lawns (125 μl, OD_600_ = 2) for 1.5 hours. CFU/worm were then quantified as above. To allow bacteria to interact with worms for a much longer time yet compare worms at a specific life stage, we further assessed established colonization in L4 stage worms exposed to microbiota bacteria from L1 stage. For established colonization, short-term persistence, and release, we raised synchronized L1 worms on bacterial lawns (125 μl, OD_600_ = 2) for 2 days. For established colonization, worms were then washed on filter-tips, transferred to M9 buffer, and CFUs extracted, and plated as above. For short-term persistence, we then washed worms on filter-tips and transferred to M9 buffer (300 μl). After taking a supernatant sample (100 μl), worms were then incubated for 1 hour (under shaking at 20°C), and a second supernatant sample (100 μl) was collected. Released bacteria were quantified as the difference between CFU in supernatant 1 and supernatant 2. CFUs maintained in worms were considered as having persisted in the short term. After 24 hours of incubation (20°C), we quantified CFU/worm as above. We would like to emphasize that our experiments aimed to control for host age, developmental stage as well as size by synchronizing worms to L4 stage in early colonization, short-term persistence, and release experiments.

### Bacterial motility on agar

Colony expansion and swarming were quantified on NGM plates with either 0.5% or 3.4% agar. For both, 0.5 μl of bacterial suspension (OD_600_ = 1) was spotted on surface-dried agar plates. After 24 and 72 hours, colony diameters were measured. Colony expansion (72 hours) and swarming (24 hours) of MYb11 mono-cultures were previously published [29].

### Simulation model of microbiota composition during a biphasic life cycle

We built a model to assess how composition of a two-member community with MYb11 and MYb71 should respond to a biphasic life cycle over time. Mirroring our evolution experiment, we consider a community that experiences 10 cycles of first associating with worm hosts, and then growing on NGM agar, and use experimentally informed parameters accordingly. We use discrete Lotka-Volterra dynamics to describe bacterial abundances in hosts and on plate. For simplicity, we call MYb11 species A and MYb71 species B. We further assume that bacterial populations always reach experimentally observed maximum population sizes (in L4 stage worms and on plates) and call these carrying capacities (on plate ${K}_P$ and in worms ${K}_W$).

Preceding the first iteration of life cycles, we assume that bacterial lawns are actively inoculated and at ${K}_P$:


(1.1) \begin{equation*} {n}_{P_A(0)}={Prop}_{A_{initial}}\ast{K}_P \end{equation*}

(1.2) \begin{equation*}\kern-.8pc {n}_{P_B(0)}={K}_P-{n}_{P_A(0)} \end{equation*}

Species abundances on plate (${n}_{P_A}$ and ${n}_{P_B}$) at time zero are thus defined by the proportion of MYb11 in the experimental inoculum (${Prop}_{A_{initial}}$) and ${K}_P$. The remaining community members are considered to be MYb71 (i.e. ${n}_{P_B(0)}$).

For subsequent cycles, we allow bacteria from the plate environment to colonize worms according to their relative fitness in worms (${w}_{W_A}$, and $(1-{w}_{W_B}$), and the carrying capacity of the host (${K}_W$) in the host-associated phase:


(2.1) \begin{equation*} {n}_{W_A\left(t+1\right)}={n}_{P_A(t)}\ast{w}_{W_A}\ast{K}_W \end{equation*}

(2.2) \begin{equation*}\kern-1.5pc {n}_{W_B\left(t+1\right)}={K}_W-{n}_{W_A(t)} \end{equation*}

We thus consider that worms are maximally colonized at every iteration, as ${K}_W$ is reached by default. This is in line with the observed consistent colonization of mono- and co-cultures. Even though the estimates for relative fitness in worms were derived from data obtained for L4 larvae, comparable proportions have been observed previously by us in adult N2 worms [13]. Therefore, the estimates used are a good approximation for the L4 to adult range.

In the free-living phase, bacteria grow in the environment according to their relative fitness on plates (${w}_{P_A}$) and ${K}_P$:


(3.1) \begin{equation*} {n}_{P_A\left(t+1\right)}={n}_{W_A(t)}\ast{w}_{P_A}\ast{K}_P \end{equation*}

(3.2) \begin{equation*} \kern-1.7pc {n}_{P_B\left(t+1\right)}={K}_P-{n}_{P_A(t)} \end{equation*}

To analyse species proportions over time, we numerically simulated 10 life cycles using the above equations in R.

To eventually compare predictions with our evolution experiment, we inferred parameter values from data collected in the ecological experiments. Specifically, we assume that relative fitness in the host is a function of persistence probability, as bacteria associated with the worm would be transferred during the experimental evolution protocol. Thus, we estimate ${w}_{W_A}$and ${w}_{W_B}$ from species proportions during short-term persistence in worms. In the evolution experiment, bacteria were then cultured on NGM for half a week. We therefore assume that ${w}_{P_A}$and ${w}_{P_B}$ is appropriately described by species proportions on agar after 3 days. Relative fitness thus always depends on the habitat-specific fitness and the influence of bacteria-bacteria interactions. As we focus on community dynamics in the absence of adaptation, we assume that fitness values are static.

### Evolution experiment

Two-member communities of MYb11 and MYb71 were serially passaged along a biphasic life cycle on NGM either with *C. elegans* (host treatment, six replicate communities) or without the host (no host control, six replicate communities). This experiment was conducted in parallel to an evolution experiment, in which MYb11 adapted host-association in mono-association with *C. elegans* [27]. Briefly, NGM agar was seeded with mixed lawns of MYb11 and MYb71 (1:1 adjustment based on OD_600_) and cultured for 3.5 days (20°C). In the host treatment, 10 L4 worms were placed on bacterial lawns and propagated until reaching the F1 generation (3.5 days). In no host controls, bacterial lawns were incubated without worms. We then collected bacteria from either worms or the agar environment at the end of every cycle. In the worm treatment, this means we collected and washed worms with M9 buffer, ground them as for the short-term persistence assay above, and transferred 10% of the community (bottleneck) the next cycle, and a sample to −80°C. Re-seeded plates had dense bacterial lawns growing with plenty of competition between founding CFUs. The no-host controls were bottlenecked using a similar number of CFUs. Specifically, we used the CFU/worm population measured in the EVO + host treatment of the previous evolutionary cycle, converted it to expected optical density in suspension (given OD = 1 is ~1.86 × 10^9^ MYb11 CFUs and ~8.0 × 10^8^ MYb71 CFUs) and adjusted bacterial suspensions accordingly. We performed 10 passages and subsequently analyzed the evolved communities.

Before phenotyping, we passaged thawed evolved communities along the evolved life cycle and grew them on NGM agar as a common garden treatment to minimize bias introduced by freezing and thawing. Subsequently, cells were collected as suspensions in M9 buffer and analyzed for growth and colonization as above.

### Genome sequencing and analysis

To assess the evolution of genetic changes, we characterized whole genome sequences of all evolved communities (n = 6) from the end of the evolution experiment (cycle 10), as well as the two ancestral strains, with which we started the experiment. The latter were included, because bacterial strains can rapidly accumulate mutations during standard maintenance, and thus, the sequencing of the particular ancestral strains used in evolution experiments facilitates the distinction of random mutations from those that spread due to selection imposed during the experiment. Total DNA was isolated from bacterial lawns using a cetyl-trimethylammonium-bromid-based protocol [31]. For HiSeq 4000 system (paired-end, 150 bp) sequencing (Illumina), libraries were prepared using the Nextera DNA Flex kit (Illumina). After quality control (FastQC v0.11.8 [32]), reads were trimmed (Trimmomatic v0.3.9 [33]). Paired reads were then aligned to the reference genomes of MYb11 and MYb71 (RefSeq: GCF_002966835.1 and RefSeq: GCF_002975205.1; Bowtie2 v2.3.3 [34]), and duplicate regions removed (Picardtools v2.22.2 [35]) using default settings. Variants differing from reference genomes were called with BCFtools v1.10.2 [36] and VarScan v2.3.9 [37], respectively. Variants called in both algorithms independently were annotated with snpEff [38, 39]. Non-synonymous variants already present in our ancestral control were filtered in R [40, 41]. Gene ontology of identified selection targets was inferred using Pseudomonas.com [42].

### Competition experiments

Competition assays were performed on plate and in worms, to measure the relative fitness of MYb11 and MYb71, and assess the impact of starting frequencies of the two species as well as the genetic host adaptation of MYb11. Specifically, relative fitness was measured free-living during growth on NGM plate, and *in vivo* during colonization and short-term persistence, as described above. To assess the impact of starting frequencies, bacterial suspensions were adjusted to varying proportions (i.e. 1:1, 10:90, 90:10, or 1:99) based on OD600. To assess the impact of genetic host adaptation, we competed ancestral MYb71 against the host specialist isolate MYb11 MT14. This MYb11 strain was previously collected [27] after experimental evolution of MYb11 (as above) in monoculture and has a frameshift mutation in the histidine kinase gene *wspE* leading to a disruption of the CheW-like domain [27], as observed for MYb11 evolved in the two-member community in the present study.

### Worm population growth

As a measure of host fitness on different bacterial lawns, we quantified worm population growth on NGM resulting from five L4 *C. elegans* after 3.5 days. As in colonization assays before, worms were maintained, washed, and collected in M9. Worm samples were frozen in 48-well plates and photographed using a Leica dissecting scope (LEICA M205 FA). We counted worms in photographs using an established image analysis pipeline [27]. Worm population sizes on MYb11 mono-cultures were previously published [27].

### Life history predictions

Maximum growth rates were predicted based on codon usage patterns using a regression model available in the *R* package gRodon 2.3.0 [43]. For the classification of life history strategies, we extended our previous approach [25] and replaced growth rate prediction by flux balance analysis with maximum growth rates from codon usage bias, because, at least theoretically, flux balance analysis is predicting rather growth yield than rate [44]. Traits were predicted for a larger microbial community [25] and given the distribution of each trait across species, a species’ trait belonging to the 0.75 (traits with high quantities) or 0.25 (traits with low quantities) quantile was considered to contribute to a strategy. Competitive traits were assumed to have high values for genome size, presence of antibiotics, and siderophore synthesis pathways, and catabolic pathways. Stress-toleration was defined by slow growth, low numbers of rRNA copies, and a high number of biofilm genes and auxotrophies. The ruderal strategy was defined by few catabolic pathways, high growth rates, rRNA copies, and codon usage bias. Pathways were predicted by gapseq 1.2 [45] using the MetaCyc pathways database [46]. Biofilm genes were inferred by abricate 1.0.1 [47] using the virulence factor database from 2023 Aug 18 [48]. The number of contributing traits was summed for each strategy, and the strategy with the highest number of contributing traits was considered a species’ final life history strategy. The scores and final assignment can be found in Supplementary Table S1.

### Statistical analysis

All statistical analyses and data plotting were performed using *R* 3.6.1 in the RStudio environment [40, 41] using ggplot2 [49]. Normality and homogeneity of variances were checked by inspecting box plots of the data and, if absent, non-parametric tests were applied.

To assess fitness differences in the host and the agar environment, we compared CFU/worm and CFU/plate between MYb11 and MYb71 with those of the community using ANOVAs followed by Dunnett post-hoc tests that corrected for multiple comparisons by false discovery rate (FDR) [50]. We tested for differences in species abundances (log10(CFU)) during co-colonization and co-growth along the biphasic life cycle using a series of paired t-tests, again corrected by FDR. To infer differences in motility between the species and the two-member community using ANOVAs and Dunnett post-hoc tests (FDR-corrected). We tested for differences in species proportions along the biphasic life cycle by comparing ancestral to evolved (EVO_+host_ and EVO_-host_) proportions using Generalized Linear Models with binomial distributions followed by Tukey post-hoc tests (FDR-corrected). To test for differences between species abundances log10(CFU) during colonization and short-term persistence of ancestral and evolved communities, we used Generalized Linear Models with Gamma distributions and Dunnett post-hoc tests (FDR-corrected). To assess the impact of varying starting frequencies on the community composition across the life cycle, we compared log10-transformed CFU numbers of the species using Mann–Whitney U tests and corrected for multiple testing using FDR. To test for differences in proportion of the ancestral and evolved MYb11 in competition, we performed paired Mann–Whitney U tests. Finally, we compared worm population sizes on different bacterial lawns or ancestral vs. evolved communities using ANOVAs with Dunnett post-hoc comparisons (FDR-corrected).

## Results

### Prediction of distinct microbial life histories for a two-member model microbiota

**Figure 1:**
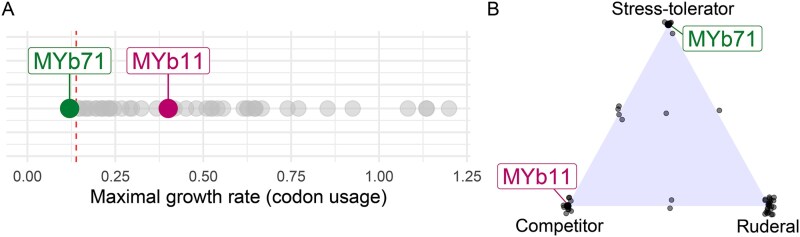
Genome characteristics of *Pseudomonas lurida* MYb11 and *Ochrobactrum vermis* MYb71 predict distinct life history strategies. (A) Inference of maximal growth rates based on codon usage patterns predicts fast growth for MYb11 (r_max_ = 0.4) and slow growth rate for MYb71 (r_max_ = 0.12). The vertical dotted line (r_max_ = 0.14) separates slow from fast-growing organisms following previous work [43]. The remaining dots show growth rates for other members of the natural *C. elegans* microbiota, for which whole genome data are available [14, 17, 25]. (B) Classification of microbiota according to Grime’s CSR framework suggests competitive and stress-tolerating strategies for MYb11 and MYb71, respectively. The remaining dots show strategies of other members of the microbiota of *C. elegans*. Points outside the corners of the triangle indicate intermediate strategies.

We inferred the life history strategies of microbiota members MYb11 and MYb71 from their genomes. Codon usage bias was previously shown to be the top genomic predictor of maximum growth rates [43, 51]. We applied the gRodon package to predict maximum growth rates from genome-wide codon usage statistics. We identified a high maximal growth rate for MYb11 (*r*_max_ = 0.4) and a low maximal growth rate for MYb71 (*r*_max_ = 0.12) (Fig. 1A). A previous codon usage bias analysis of over 200 000 organisms revealed a bimodal distribution of inferred growth patterns, with a threshold of 5 hours doubling time that separates slow from fast growth [43], now suggesting that MYb11 (inferred doubling time of 1.7 hours) falls into the group of fast growing organisms, whereas MYb71 (doubling time of 5.8 hours) belongs to that of slow growing organisms. Previous work further revealed that growth rate is positively linked to high codon usage bias of highly expressed ribosomal proteins [52]. In our case, we indeed found a substantially stronger codon usage bias for these proteins in MYb11 (CUBHE = 0.86) compared to MYb71 (CUBHE = 0.67).

We further characterized bacterial life-history strategies by applying the widely used framework from Grime that conceptualizes a three-way tradeoff between competitive, stress-tolerant, and ruderal strategies, whose success varies depending on disturbance and stress levels [53–55]. This framework was shown to have high explanatory power of the diversity of life-history strategies in comparison to other frameworks [53, 54]; it was successfully adapted to microbial communities in the past [53, 56]; and we previously used it to predict microbial life history strategies from genome characteristics for two synthetic *C. elegans*–associated microbiota communities [14, 25]. Based on this approach, we found distinct strategies for the two microbiota members, with MYb11 classified as a competitor and MYb71 as stress-tolerant (Fig. 1B, Supplementary Table S1). Together, predicted differences in growth and ecological adaptation suggest opposite microbial life history strategies for the two-member microbiota. As the different microbial strategies were previously associated with varying success in host colonization [14], we next specifically characterized bacterial fitness in the host and its environment using an experimental approach.

### Habitat context determines interspecies interactions and bacterial fitness

**Figure 2:**
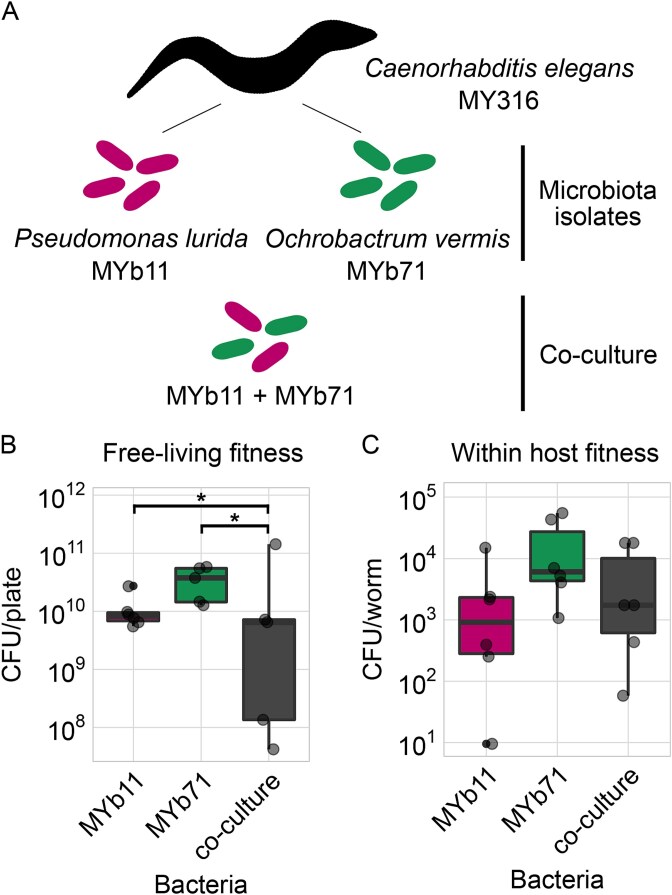
Microbiota members MYb11 and MYb71 compete in the free-living environment, but apparently not within the host. (A) The model microbiota community used in this study consists of MYb11 and MYb71, and is investigated in their natural host, *C. elegans* strain MY316, in mono-culture and in co-culture (always combined in equal proportions based on OD600). (B) Free-living fitness of bacteria was measured as CFUs that grew on NGM within 72 hours of incubation at 20°C (equivalent to selection time point of the later evolution experiment, Fig. 4). (C) Within-host fitness was measured as CFU/worm collected from *C. elegans* worms raised on the respective bacteria. (B, C) Replicates are shown as individual data points (5 ≤ n ≤ 6). Box plots show median (center line), upper and lower quartiles (box limits), and the interquartile range (whiskers). Differences between monocultures and the co-culture were assessed using Dunnett tests (^*^ = *P* < .05).

To assess bacterial fitness of microbiota isolates MYb11 and MYb71 in the host and the free-living environment (Fig. 2A), we quantified colony forming units of both species in mono- and co-association in the two habitats. In the free-living agar environment, they both showed significantly greater population sizes after 3 days growth on NGM in monoculture than together (Fig. 2B, Supplementary Table S2), indicating competition. We selected this time point as a reference for free-living fitness, as it reflects the duration of the free-living phase in our evolution experiment below. As expected based on their co-isolation from a natural worm isolate (i.e. *C. elegans* strain MY316) [12], both species colonized nematodes alone and in combination. Within worms, however, competition was attenuated, as they reached comparable abundances that were not significantly different between mono- and co-colonization (Fig. 2C, Supplementary Table S3). We conclude that the environmental habitat, but apparently not the host habitat, induces strong competition among strains and thus appears to represent a habitat that may exert strong selection on the bacteria. As habitat appeared to shape bacterial fitness and interactions, we assessed bacterial fitness along the life cycle in greater detail.

### Microbiota members vary in fitness along the biphasic life cycle

**Figure 3:**
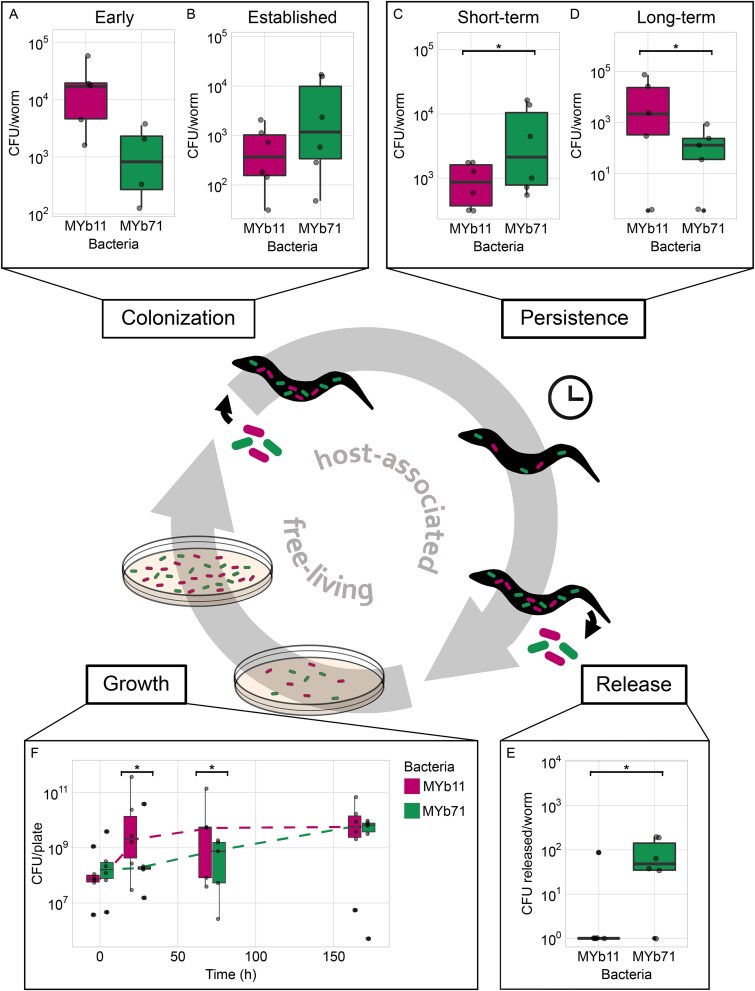
Microbiota members MYb11 and MYb71 vary in fitness along the biphasic symbiotic life cycle. Bacterial fitness was measured during co-colonization of hosts (CFU per bacterial strain and worm) or as co-cultures under free-living conditions (CFU per bacterial strain per population on nematode growth agar, NGM). The illustration in the middle indicates the biphasic life cycle and its direction, as implemented in our experiment. It further indicates the approximate points along the life cycle, which are covered by the independently measured traits. The host-associated bacterial traits include (A) early colonization of fourth larval instar (L4) worms (within 1.5-hour exposure), (B) established colonization (i.e. colonization upon long-term exposure, similarly using L4 worms that however developed on respective bacteria since the first larval stage; co-culture from Fig. 1C), (C) short-term persistence in worms (bacterial maintenance within worms after 1 hour in a bacteria-free M9 medium stressful for the nematodes), and (D) release from L4 stage worms into buffer within 1 hour (measured in parallel to persistence). (E) Free-living fitness (i.e. growth on NGM) in the co-culture was assessed over 3 days and sampled at 0, 24, and 72 hours. Dashed lines connect median CFU/plate-values between time points. The relative frequencies of the two strains in co-culture was inferred by plating bacteria on species-specific selective media. (A–E) Replicates are shown as individual data points (4 ≤ n ≤ 6). Box plots show median (center line), upper and lower quartiles (box limits), and the interquartile range (whiskers). Differences between the bacterial species’ abundances were evaluated using paired t-tests (^*^ = *P* < .05). Bacteria grew on plates as dense lawns, individual bacteria shown are a symbolic illustration of the two species co-occurring.

In a biphasic life cycle, symbionts enter hosts, establish and persist, and are released back into the environment, where they may proliferate (Fig. 3). To compare variation in life history strategies between MYb11 and MYb71, we performed competition experiments along the life cycle using species abundances (colony forming units) as a proxy for fitness and, thus, as a relevant measure that determines the bacteria’s performance within the two-member model community during a single as well as across several biphasic life cycles (see also [13, 30, 57]). In the free-living environment, our two focal species showed distinct growth dynamics. With the later evolution experiment in mind, we compared their growth on NGM agar over several days. After 24 and 72 hours, MYb11 was significantly more abundant on agar (Fig. 3E; Supplementary Table S4). This revealed MYb11 as a better competitor during early and mid-term growth on NGM, where both species suffer a fitness cost when together (Fig. 2B). Furthermore, we observed a significantly higher motility of MYb11 compared to MYb71 (Fig. S1; Supplementary Table S5). Even though these latter results are not directly transferrable to the agar plates used for studying the biphasic life cycle, even an only minor advantage in motility could still contribute to an enhanced access to resources and thus competitiveness for MYb11 relative to MYb71.

We assessed relative bacterial frequencies along the host-associated part of the life cycle. As bacteria are continuously taken up orally by *C. elegans*, their abundance within the host is naturally a consequence of both, bacterial competitiveness on agar plates (from which bacteria are taken up) and bacterial competitiveness in the host environment. We assessed early colonization, later established colonization, and also later persistence, where bacteria face different challenges, including being taken up by the worm and passing the grinder organ in the pharynx (especially early colonization), being maintained in the gut through adhesion or biofilm formation (all three phases, but especially persistence in a bacteria-free and thus stressful environment for the worm that should activate diverse nematode stress responses), survival of digestive enzymes and an activated immune response (all three phases, especially persistence as above), competition with other bacteria for entry (especially early colonization), and access to space and nutrients (all three phases, especially the latter two). In the early phase of colonization (i.e. 1.5 hours after worm exposure), similar numbers of MYb11 and MYb71 were found in the *C. elegans* host, although often MYb11 dominated (Fig. 3A; Supplementary Table S4). In the established stage of colonization (i.e. after long-term exposure of hosts to the bacteria), we found species composition to be roughly even, although MYb71 dominated more often than MYb11 (Fig. 3B; Supplementary Table S4). Next, we assessed more stringent persistence in worms. Specifically, we measured short-term persistence as the number of bacteria that are not immediately excreted or digested within 1 hour of worms being away from the environmental symbiont pool. After 1 hour in buffer, MYb71 was significantly more abundant, suggesting its higher competitiveness within the worm host (Fig. 3C; Supplementary Table S4). Because the persistence assay is performed in an initially bacteria-free environment, it additionally allowed us to quantify bacteria that exited the worms as a proxy for bacterial release, even if bacterial numbers may be low in L4 nematodes. In turn, such bacterial release can affect bacterial numbers for the subsequent phase of this part of the life cycle. Using this approach, we observed a greater abundance of MYb71 (Fig. 3D, Supplementary Table S4), suggesting that MYb71 both accumulates in worms and is efficiently released from the host.

Together, our results indicate that a rapid onset of growth (on NGM) provides a competitive advantage to MYb11 in the environmental habitat on plates, whereas MYb71 appears to have an advantage in the host because of its better ability to persist and subsequently be released from *C. elegans*. Based on these results, we next asked to what extent these differences in MYb11 and MYb71 life history strategy influence the relative abundance and also evolutionary adaptation of the two bacteria to the bi-phasic life cycle during an evolution experiment.

### Initial differences in life history strategy influence microbial abundance and adaptation to a host-associated life cycle

**Figure 4:**
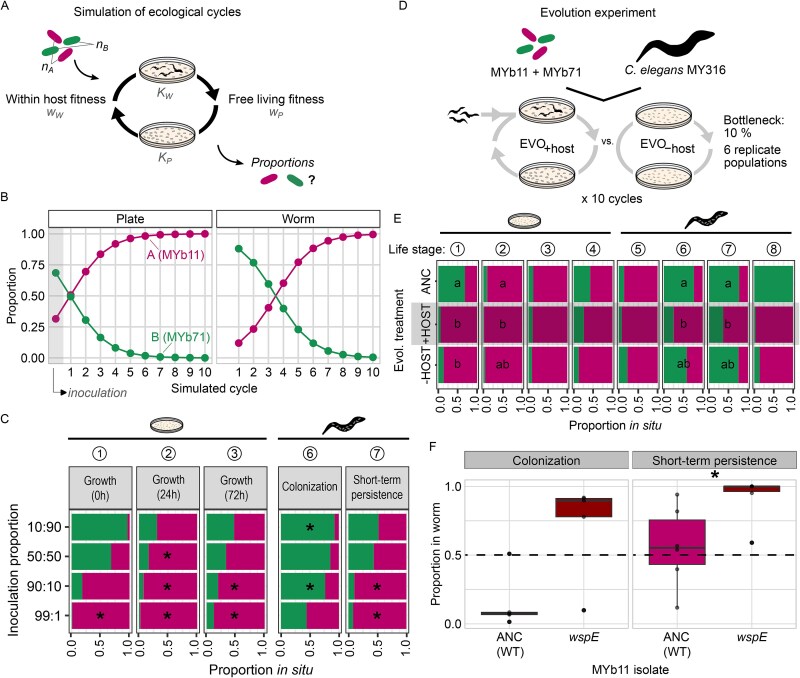
Repeated iterations of a biphasic life cycle led to enrichment and host adaptation of the better environmental competitor MYb11. (A) Mathematical model of a simplified symbiotic life cycle was used to predict species proportions within the microbiota over eco-evolutionary time. Fitness within the host or free-living environment and the carrying capacities of both habitats were estimated from empirical data (Fig. 3). (B) Predicted species proportions across 10 iterations of the simulated symbiotic life cycle in the free-living environment (plate) and the directly connected host environment (worm). (C) To test the main prediction of the mathematical model, species starting frequencies (i.e. 10:90, 50:50, 90:10, 99:10 for MYb11:MYb71) were varied experimentally, and relative proportions in the community measured along the life cycle. The circled numbers in the top row are the same as in panel E, facilitating comparison of results for specific parts of the life cycle as a consequence of either ecological dynamics (here) or evolutionary dynamics (panel E). (D) In an evolution experiment, the two-member community of MYb11 and MYb71 was passaged either in a symbiotic biphasic life cycle with *C. elegans* as a host (EVO_+host_) or in a control treatment without hosts, but otherwise identical conditions. The composition of ancestral and evolved microbiota communities from the end of the evolution experiment was compared along the stages of the symbiotic life cycle. (E) Final proportions of species in ancestral communities as well as communities from the end of the EVO_+host_ and EVO_–host_ evolution experiments, which were subjected to one complete biphasic life cycle with the host, in all cases with an initial 1:1 ratio of the two species at the start of the life cycle. Median species proportions for growth on NGM plates at [1] 0, [2] 24, [3] 72, and [4] 168 hours, as well as in worms during [5] early colonization, [6] colonization, [7] short-term persistence, and [8] release are shown. (F) The relative fitness of an evolved, genetically adapted host-specialist MYb11 was tested against MYb71 during colonization and short-term persistence in worms (identical starting frequencies of each). Experiments were performed in independent biological replicates respectively (5–6 replicates). Statistical differences in species proportions between the ancestral and evolved communities are indicated by letters (generalized linear model with a binomial distribution and Tukey post-hoc tests, Fig. S3). Statistical differences in species abundances (MYb11 vs. MYb71 in C) or proportions (proportion ancestral vs. evolved in F) were computed using (paired for F) Mann–Whitney U tests and corrected for multiple testing using FDR (^*^P ≤ .05).

To evaluate whether the presence of distinct life history strategies influences microbiota composition, we first used a mathematical model to simulate the ecological dynamics of the two-member community across the biphasic life cycle over time. Abstracting the biphasic life cycle in a discrete mathematical model inspired by Lotka-Volterra dynamics, we focused on the two key selective pressures in the host and the environment: persistence in worms and growth on agar (Fig. 4A; see equations 1.1–3.2 in the Methods), and parameterized it with our experimental data. Specifically, we simulated a two-member community initially starting with both species as in our ecological experiments (Methods; Supplementary Table S6). Bacteria from this community could then enter and persist in worms up to a maximum carrying capacity (${K}_W$). Species abundances were weighted according to the relative fitness in worms (${w}_W$; Methods; Supplementary Table S6). This worm-associated community would then seed the connected environmental habitat according to a plate-specific carrying capacity (${K}_P$) and the free-living fitness of the species (${w}_P$), thereby determining the relative proportions of the two species over time. Simulating 10 iterations of the life cycle, we observed that MYb11 rapidly increased in proportion in the environment and nearly outcompeted MYb71 by six cycles (Fig. 4B). In simulated worms, we observed similar, yet lagging dynamics. Here too, MYb11 increased over time, dominated the microbiota after only four cycles, and subsequently approached fixation (Fig. 4B). In the absence of evolutionary adaptation, this suggests that MYb11 may enrich in worms when it outnumbers MYb71 in the environment. To test this hypothesis, we performed competition experiments on plates and in hosts with different starting frequencies of the two species (Fig. 4C), thereby simulating one round of the simulations. Both on plates and in worms we observed a greater proportion of MYb11 the more common it was in the inoculum. This was most pronounced when MYb11 was inoculated in excess (i.e. at 90%–99% of the inoculum; Fig. 4C, Supplementary Table S7). Nevertheless, as observed above (Fig. 3), MYb71 showed higher abundance in the host than expected from the initial proportions on plates, especially for our measure of colonization and sometimes also for short-term persistence when MYb71 initial frequency was 10% or 1% (the 90:10 and 99:1 treatments, Fig. 4C). In conclusion, the results of the mathematical model suggest that the numeric advantage in the environmental microbial pool can influence species proportions in the host-associated community, whereas the experimental results indicate involvement of an additional factor in determining species composition in the host.

To empirically test and further explore the results from the simulations, we performed an evolution experiment with the two-member community in a biphasic life cycle with a host-associated phase (EVO_+host_) and compared this to a control without hosts but otherwise identical conditions (EVO_-host_; Fig. 4D), generally following our previously developed approach [27]. Both treatments were carried out in parallel and under identical conditions. The only difference was that for the EVO_+host_ treatment, hosts were added to inoculated agar plates after 3.5 days of a particular cycle, followed by isolation of bacteria from the colonized nematodes at the end of each cycle (Day 7), whereas the EVO_-host_ treatment did not include any hosts, and bacteria were washed off plates at the end of a particular cycle. For the EVO_+host_ treatment, 10% of the obtained bacteria at the end of a cycle were used to inoculate the agar plate for the next cycle, whereas for the EVO_-host_ treatment we used a comparable number of bacteria for inoculation. In the EVO_+host_ treatment, freshly prepared hosts of the same *C. elegans* strain MY316, always thawed from frozen stock cultures, were used, thus ensuring that the factor host is constant across the entire experiment. During this experiment, possible changes in bacterial frequencies can be driven by ecological dynamics, as simulated above with the mathematical model. Additionally, genetic changes in the bacterial lineages can drive evolutionary changes in microbiota members. After serially passaging six independent replicate communities across 10 cycles, we characterized fitness of the bacteria from the end of the experiment as well as ancestral bacteria, in all cases along the key phases of the life cycle. As predicted, MYb11 dominated the final communities on agar from early on, even more strongly than observed for the ancestral community (Fig. 4E; Fig. S2; Supplementary Table S8). Given our simulations, we expected that the growth advantage of MYb11 on NGM should lead to a particularly strong enrichment in the environmental habitat, as observed, but initially not within the host. Moreover, evolved MYb11 also showed significantly higher fitness during worm colonization and persistence (Fig. 4E; Fig. S2; Supplementary Table S8). This proportion was higher than that found for the same parts of the life cycle under pure ecological dynamics in Fig. 4C, possibly indicating an adaptation of the evolving MYb11 bacteria to an association with hosts.

To assess whether MYb11’s apparent advantage in worms came at a cost to MYb71, we determined their absolute abundances in the worm. Comparing the number of MYb71 CFUs in ancestral and the final communities, however, showed that MYb71 abundance in worms remained stable during colonization, and possibly increased during short-term persistence (Fig. S3; Supplementary Table S9). Thus, the relative increase of MYb11 in hosts appears to be due to its evolutionary adaptation to the host environment rather than improved direct competition with MYb71.

To assess any evolutionary changes in the bacterial lineages, we analyzed whole genome sequences of entire bacterial communities of four replicate populations per treatment from the end of evolution, which were mapped on available reference genomes for ancestral MYb11 and MYb71 [17], followed by inference of genetic variants that differed between ancestral and the evolved bacteria. Briefly, we found numerous non-silent substitutions and indels in different genes of evolved MYb11, including mutations in two genes of the *wsp* operon: *wspE* and *wspF* (Supplementary Table S10), which both influence activity of the second messenger c-di-GMP that itself acts as a regulator of numerous life history traits, e.g. wrinkly colony morphology or biofilm formation [58]. This is in line with the appearance of wrinkly colony morphologies, previously linked to *wsp* operon mutations in MYb11 and an increase in persistence within the host [28]. In contrast, we detected no difference between the ancestral and final MYb71 genomes (Supplementary Table S10), highlighting that at the genetic level only MYb11 shows signatures of adaptation.

To test a possible selective advantage of evolved MYb11, we performed competition experiments in worms between MYb71 and either the ancestral MYb11 or, alternatively, a previously isolated host specialist MYb11 with a frameshift mutation in *wspE* [27]. The host specialist MYb11 mutant indeed showed a higher competitive advantage during short-term persistence (Fig. 4F; paired Mann–Whitney U test with *U* = 0, *P* = .03), yet not during colonization alongside MYb71 (Fig. 4F); paired Mann–Whitney U test with *U* = 1, *P* = .13).

Together, the combination of simulation and evolution experiments revealed that the MYb11 growth advantage in the NGM plate environment translates into an enrichment within hosts over ecological time. Moreover, this advantage was further boosted by genetic adaption to the host over evolutionary time. We next wondered whether the observed evolutionary changes in microbiota composition have an effect on the *C. elegans* hosts.

### Community evolution influences microbiota impact on host fitness

**Figure 5:**
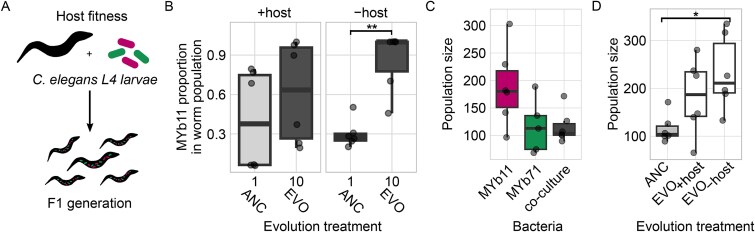
Microbiota composition and evolution affect host fitness. (A) Host fitness (i.e. worm population size) was quantified in association with bacterial mono- and co-cultures. For this, the number of worms in the F1 generation produced by five *C. elegans* MY316 L4 stage within 3.5 days was counted. Replicate worm populations are shown as individual data points. (B) Proportions of MYb11 in the ancestral and evolved communities during colonization of worm populations were compared using Mann–Whitney U tests. Differences between (C) worm population sizes on ancestral mono- and co-cultures of MYb11 and MYb71, as well as differences between (D) worm population sizes of ancestral and evolved communities (EVO_+host_ and EVO_–host_) were determined using ANOVAs with Dunnett post hoc comparisons (^*^P < .05). Population size on the Y axis of panels C and D is given as the number of worms. The experimental evolution treatments (X axis in panels B and D) included evolution of microbial communities in either presence (+host) or absence (−host) of *C. elegans* hosts.

When the composition of the microbiota varies or when individual microbes adapt to host association, then this can lead to changes in the microbes’ interaction with the host, including detrimental interactions if the microbes evolve to exploit the host. Therefore, we examined the fitness of *C. elegans* at the level of the worm population upon colonization with ancestral or evolved bacteria (Fig. 5A). Furthermore, we confirmed that at the level of the worm population, colonization of ancestral and evolved communities mirrored that in individual worms (Fig. 4E, Fig. 5B): a subtle, albeit not statistically indistinguishable higher median proportion of MYb11 after evolution with the host (Mann–Whitney U test: *U* = 9, *P* = .2), and a significant increase in MYb11 proportion after evolution on plates (Mann–Whitney U test: *U* = 1, *P* = .007). For worms colonized with ancestral species or communities, we found no statistical differences, although the F1 generation’s population size on the co-culture resembled that of MYb71 more than that of MYb11 (Fig. 5C; Supplementary Table S11). Comparing host fitness on ancestral and evolved communities, we observed that worms maintained on evolved co-cultures had slightly greater population sizes than those on ancestral bacteria (Fig. 5D, Supplementary Table S11). Consistent with worms reaching the highest population sizes in the presence of MYb11 (Fig. 5C), we found that worms on evolved, MYb11-enriched evolved microbiotas could reach greater population sizes than on the ancestral community (Fig. 5D). However, this was only significant for worms on EVO_-host_ communities, whereas we found no significant differences between worms in EVO_-host_ and EVO_+host_ communities (Fig. 5D, Supplementary Table S11). These results show that the observed changes in community composition and possibly any evolutionary changes in MYb11 on plates, but apparently not in hosts, produced a beneficial effect on host fitness. The effect was clearly not detrimental, which could have been possible, considering that MYb11 is able to express a pathogenic phenotype under certain conditions [15, 59, 60]. Overall, our results highlight that eco-evolutionary changes in the microbiota can feed back on host fitness.

## Discussion

In this study, we investigated the role of distinct microbial life history strategies on subsequent adaptation to, and interaction with a host. Starting with genomic evidence indicating two distinct life history strategies, we performed colonization, growth, and evolution experiments along a biphasic life cycle with a host-associated and a free-living phase utilizing a two-member model community of *P. lurida* MYb11 and *O. vermis* MYb71 from the natural microbiota of *C. elegans.* Here, we focused on two ecologically relevant microbial life history strategies: a high environmental, but low within-host competitor (i.e. strategy expressed by MYb11) versus a low environmental, but high within-host specialist (i.e. expressed by MYb71). Our results show that the better environmental competitor, MYb11, adapts well to the host-associated life cycle and, in the end, it even shows higher abundance than the initially better within-host specialist, MYb71. A likely reason is that competition among strains is stronger in the environmental habitat but apparently absent in the host habitat (Fig. 2). In turn, the initial numeric advantage of MYb11 in the environmental pool as well as further evolutionary adaptation overrides the initial fitness advantage of MYb71 within the host (Fig. 4). Intriguingly, the resulting change in microbiota composition affects host fitness, as population sizes of *C. elegans* was higher when in association with evolved rather than ancestral microbiota. Taken together, our findings highlight the particular role of variation in life history strategies in shaping microbial adaptation to the host-associated life cycle and the resulting fitness effects on the host. These conclusions are based on the studied two-member model community. As host-associated microbiomes commonly consist of more complex communities with diverse interactions possible among community members, it will be particularly insightful to experimentally assess our findings in more diverse communities in the future. In the following, we will use our current results to first discuss the presence of distinct life history strategies within host-associated microbial communities. We then focus on how niche partitioning maintains species co-existence along the biphasic life cycle. Finally, we analyze how adaptation of an environment-centric microbe to host association enhances microbial fitness in the host.

Microbial taxa that are reliably found in association with a specific animal or plant, may be adapted to their hosts or are simply present, because they are abundant in the hosts’ environment. In this study, we showed that the co-occurring bacteria vary in their fitness across the biphasic life cycle, whereby MYb11 has an advantage in the free-living environment and MYb71 in the host. This variation is also reflected by differences in life-history characteristics that we here inferred from genome sequence data, indicating comparatively high maximum growth rates together with competitive traits for MYb11, and comparatively low maximum growth rates and rather stress-tolerating traits for MYb71. Our previous study with a more complex 43-member microbiota community of *C. elegans*, already assessed the relationship between genome-inferred life-history traits and microbial interaction with a host, and similarly indicated that the MYb11-associated traits (i.e. fast growth, competitive traits) should favor growth outside of a host, whereas those associated with MYb71 (low growth rates, stress tolerance) should favor host colonization [25]. These inferences are consistent with several independent, previous analyses of the *C. elegans* microbiota, performed by us and also other groups using more complex microbial communities, uniformly demonstrating a much stronger general enrichment of the *O. vermis* isolate MYb71 and also related *Ochrobactrum* strains in worms under a variety of experimental conditions [17, 21, 23, 25]. In some cases, this has been linked to host insulin signaling [21], however, different *Ochrobactrum* strains seem generally able to colonize nematodes, as they can also be found in the microbiota of predatory *Pristionchus* nematodes [61] and as secondary symbionts of entomopathogenic nematodes [62]. In contrast, *P. lurida* MYb11 was more competitive in the agar environment, reflecting an environmental advantage. Although pseudomonads are considered a core taxon of the natural *C. elegans* microbiota [18, 22, 29], they are usually present at low abundance both in natural and model *C. elegans* microbiota communities [12, 17, 22], and are widely spread across environments, and considered one of the most versatile bacterial genera [63]. Moreover, both bacterial species co-existed within worms, which might itself be stabilized by the distinct life history strategies [64]. Together, this highlights that distinct life history strategies can succeed along a biphasic lifecycle.

Although host- and environment-centric strategies can coexist, we observed that the initially better environmental competitor outcompetes the initial host specialist upon experimental evolution across a host-associated life cycle. This is in agreement with a recent theoretical model of microbes following a biphasic life cycle, which suggests that microbes may use two alternative strategies to optimize fitness overall [10]. Either microbes invest in increased replication in the environment or, alternatively, they decrease migration, thereby reaping the benefits of rapid growth in the environment (i.e. the first optimal strategy). The resulting increase in the symbiont source pool increases transmission to novel hosts, which has been shown to even overwhelm selection by host immunity in zebrafish [65]. Our data demonstrate that a high abundance in the source pool (as observed for MYb11) may be key for achieving high abundance within hosts as well as adapting to the specific host environment, thereby countering a possible prior advantage by initially present host specialist bacteria (e.g. MYb71). Thus, inter-host dynamics, including growth and survival in the free-living environment, substantially affect within-host microbiota assembly.

Over evolutionary time scales, microbiota bacteria can further adapt to their hosts. We observed that environment-centric MYb11 increased its abundance in communities serially passaged in the presence of hosts, even more so than those which were passaged on agar alone. This confirms that species sorting as well as genetic adaptation shape the characteristics of this host-associated microbiota. In line with the appearance of wrinkly MYb11 colonies, which were previously described as *C. elegans* host specialists [27], we detected mutations in two genes of the *wsp* operon in the EVO_+host_ communities. We could further validate the selective advantage of host specialist MYb11 that mutated the CheW-like domain of the histidine kinase WspE. This suggests that similar to MYb11 adapting to *C. elegans* in mono-association, its adaptation to the host as member of a model community is also linked to the wrinkly spreader (Wsp) system, a two-component system that pseudomonads use to integrate extracellular signals (e.g. surface sensing) with the help of the second messenger c-di-GMP to adjust bacterial behavior accordingly [58, 66, 67]. This finding underscores the key role of c-di-GMP in host-association, which can be further explored in the future with the help genetic mutants with either no, reduced, or alternatively increased c-di-GMP activation. It also suggests that *Pseudomonas’* adaptive strategies could follow similar evolutionary trajectories in more complex host-associated microbial communities, as reported for natural *C. elegans* isolates [12, 18, 22], clearly deserving further evaluation with more diverse microbiota associations in the future.

Although MYb11 evidently evolved during experimental evolution, we did not detect any mutations in evolved MYb71. Even though this might highlight how well this bacterium was initially able to colonize the host, the lack of mutations could also be explained by limited access to mutations as MYb71 proportions decreased within the microbiota, which is likely associated with a smaller population size and thus a lower probability of favorable mutations to occur, or simply by a lower mutation rate. In genomic analysis, we found a higher codon usage bias of highly expressed ribosomal proteins in MYb11 compared to MYb71, which is typical for organisms adapting to multiple environments [52]. Together with the higher abundance in growth experiments and the genomic adaptation of MYb11 during the evolution experiment suggest that MYb11 indeed has a greater evolutionary potential than MYb71. Taken together, we conclude that host adaptation of an environmental competitor, MYb11, not only provides an advantage in mono-association with the host but is robust in the context of a two-member community.

Despite fluctuations along the biphasic life cycle, both species were maintained in our model system over ecological and evolutionary time scales, albeit at different abundances. Although extending the evolution experiment might have allowed MYb11 to exclude MYb71 completely, co-existence of the two species was expected, as they have been co-isolated from the *C. elegans* strain MY316 collected in nature, they have been co-cultured in *C. elegans* and on agar before, and can be maintained within a more complex microbiota [12, 13, 17]. Classically, such patterns of co-occurrence are often explained by niche partitioning as highly similar species should exclude each other by competition over time [68]. There are two reasons why niche partitioning along the biphasic life cycle could explain coexistence in our system. Firstly, we started with both a host-centric and an environment-centric microbe, thus our system already had distinct favored niches. It is then possible that both habitats can, at least temporarily, serve as a reservoir for either of the species if either has a strong advantage when rare. Secondly, additional niche partitioning is likely to occur within the host. As we cannot link the observed genetic changes to a change in interaction between MYb11 and MYb71, it is possible that they do not directly compete within the host. Similar to our observations here, the two main colonizers of the fresh water polyp *Hydra*, the bacteria *Duganella* and *Curvibacter*, only stably co-exist on hosts, whereas *Duganella* outcompetes *Curvibacter in vitro* [69]. Distinct within-host niches might attenuate competition and allow maintenance of MYb71 despite overwhelming MYb11 influx and host adaptation, which could be addressed in future studies.

In addition to niche specialization within the host habitat, we saw variation in microbiota composition over time. Given that we started experiments with non-colonized, sterile worms, we may consider this temporal variation a succession series. Our data suggest that MYb11 initially enters worms efficiently, yet decreases in relative abundance as MYb71 accumulates over time. Similar successions of microbial colonizers have also been observed in other hosts, e.g. in the house mouse [70]. The differential abundance of MYb11 and MYb71 may additionally be shaped by host responses to the two bacteria. The gene expression response towards either MYb11 or MYb71 has been characterized before in separate studies, yielding some overlap in the inducible genes, e.g. the downregulation of the acyl-CoA-dehydrogenase ACDH-1 or the general activation of C-type lectins [20, 71]. The two bacteria also elicit distinct responses, including activation of different components of the nematode’s immune system, such as insulin-like signaling by MYb71 or targets of the p38 MAPK pathway by MYb11. Intriguingly, *Ochrobactrum* bacteria fare well upon activation of insulin-like signaling [21], whereas MYb11 abundance is reduced under these conditions or in the presence of an intact p38 MAPK pathway [59]. Our current results suggest that such host responses could have affected bacterial abundances initially (i.e. MYb11 became initially less abundant within worms; Fig. 3B, C), but less so at later time-points of the evolution experiment when MYb11 also dominated the host-associated part of the life cycle (Fig. 4).

The observed change in community composition could also translate to a changed impact on *C. elegans* traits, especially in consideration of the known differences in the bacteria’s functional repertoires [14]. Indeed, hosts associated with the evolved, MYb11-enriched microbiota, reached greater population sizes than those on the ancestral community (Fig. 5). A possible explanation for this finding is that MYb11 but not MYb71 can produce thiamine and pantothenate [14], which are both essential vitamins for *C. elegans* [72]. Moreover, thiamine can also attenuate mitochondrial stress in a *C. elegans* disease model of dihydrolipoamide dehydrogenase deficiency [73], thus providing additional benefits. We hypothesize that species sorting led to changes in host fitness, as we did not observe any genetic mutations that should affect synthesis of thiamine or pantothenate or any known beneficial function in the host-adapted MYb11. The particular role of vitamin production for microbiome-mediated effects on host fitness warrants further evaluation in the future, e.g. using mutants with altered vitamin biosynthesis or vitamin supplementation experiments.

## Conclusions

Our study demonstrates that the presence of distinct microbial life history strategies influences microbiota characteristics and also further adaptation of individual microbes to host association. Our study moreover highlights that eco-evolutionary dynamics in the symbiont source community directly feed back to the host-associated microbiota and can ultimately shape host fitness. In turn, the explicit consideration of microbial life history strategies and competition levels across the host-associated life cycle is key for a more differentiated understanding of the eco-evolutionary dynamics in microbiota communities and their resulting functional effects on the host.

## Supplementary Material

Supplementary_Figures_Obeng_etal_wraf168

Supplementary_Tables_Obeng_etal_wraf168

## Data Availability

Raw sequencing data are available in the NCBI Bioproject (PRJNA1293856). All other data are accessible alongside custom code via https://github.com/nobeng/MYb11-MYb71-community (release archived at Zenodo, DOI: 10.5281/zenodo.16360349).

## Code availability

Custom code is available via https://github.com/nobeng/MYb11-MYb71-community (release archived at Zenodo, DOI: 10.5281/zenodo.16360349).
