# Supplementary material for: Role of microbial life history strategy in shaping the characteristics and evolution of host-microbiota interactions
Source: ISME J. 2025 Aug 5;19(1):wraf168. doi: 10.1093/ismejo/wraf168 (PMC12404661; doi:10.1093/ismejo/wraf168)
Supplement: Supplementary_Figures_Obeng_etal_wraf168 [file supplementary_figures_obeng_etal_wraf168.pdf]

Supplementary Figures

Full title: **Role of microbial life history strategy in shaping the characteristics and evolution of host-microbiota interactions**

Short title: Microbe life history shapes host interactions

Authors: Nancy Obeng[1,2], Johannes Zimmermann[1,3,4], Anna Czerwinski[1], Janina Fuß[5], Hinrich Schulenburg[1,3]

Affiliations:

[1]Department of Evolutionary Ecology and Genetics, University of Kiel; 24118 Kiel, Germany.

[2]Present address: Roche Pharma Research and Early Development, Infectious Disease, Roche Innovation Center Basel, F. Hoffmann-La Roche, Basel, Switzerland.

[3]Max Planck Institute for Evolutionary Biology; 24306 Plön, Germany

[4]Cluster of Excellence Balance of the Microverse, Friedrich Schiller University, 07745 Jena, Germany

[5]Institute of Clinical Molecular Biology, University of Kiel; 24118 Kiel, Germany.

Corresponding authors: N. Obeng (nobeng@zoologie.uni-kiel.de), H. Schulenburg (hschulenburg@zoologie.uni-kiel.de)

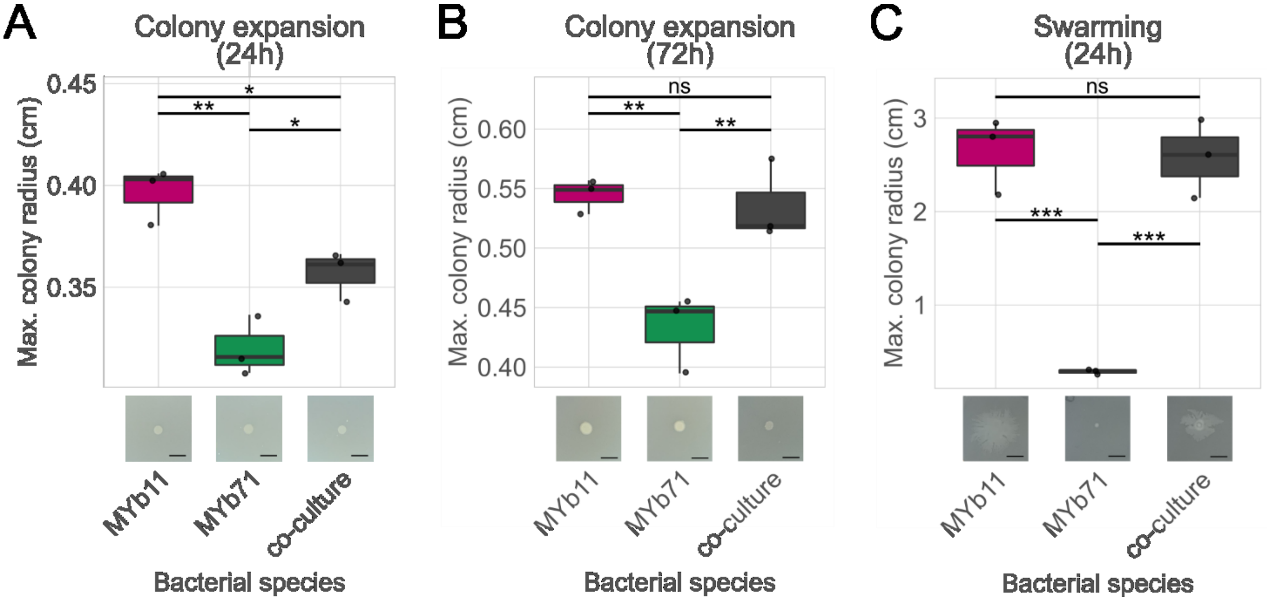

Figure S1. Motility of *Pseudomonas lurida* MYb11, *Ochrobactrum vermis* MYb71 and a co-culture of the two on nematode growth agar. A, B, Colony expansion on 3.4% agar was measured as maximum colony radius after 24h and, B, 72 hours after spotting. **C,** Swarming motility was assayed on 0.5% agar, and max. colony diameter measured after 24h. Technical replicates are shown as individual data points. Species were compared using ANOVAs followed by Tukey post-hoc tests (***= $P < 0.001$, ** = $P < 0.01$, * = $P < 0.05$). Images of representative replicates are shown along the x-axis, scale bars = 0.5 cm.

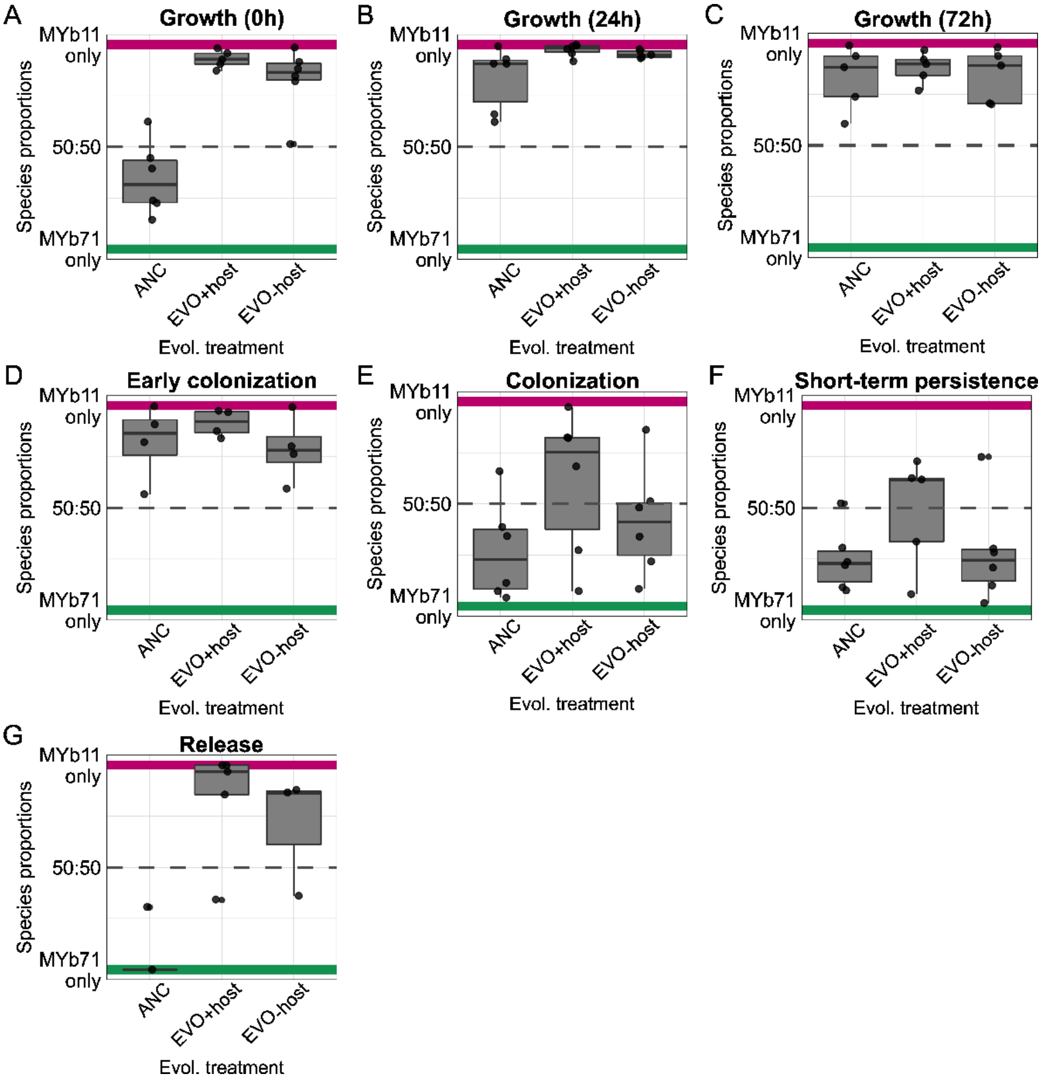

Figure S2. Proportions of *Pseudomonas lurida* MYb11 and *Ochrobactrum vermis* MYb71 in ancestral and evolved co-cultures across the stages of the biphasic life cycle. Species proportions were quantified at, **A**, inoculation of bacterial lawns (0h), **B**, after growth on nematode growth agar for 24h, and **C**, 72h. In host association, species proportions were quantified during: **D**, during early colonization of L4 stage *C. elegans* (after 1.5h bacterial exposure), **E**, established colonization of L4 stage worms (exposed to bacteria since L1), **F,** short-term persistence in colonized L4 stage worms kept in buffer for 1h, and **G,** release L4 stage worms into buffer within 1h. Replicate co-cultures are depicted as individual data points, dashed lines indicate even species proportions.

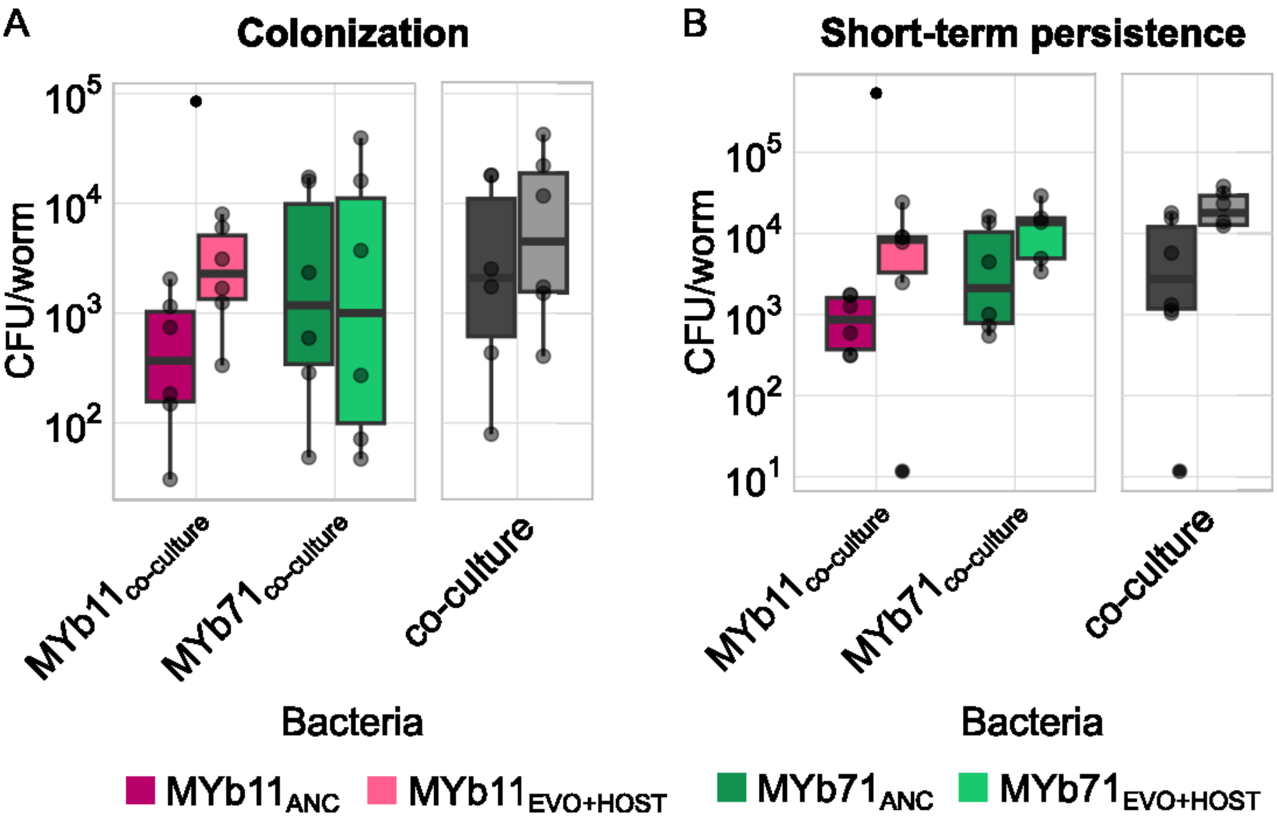

Figure S3. Microbiota composition during colonization and short-term persistence of ancestral and evolved co-cultures of MYb11 and MYb71. A, Colonization is shown as CFU/worm in L4 stage *C. elegans* raised on an ancestral or host-evolved (EVO_{+host}) co-cultures. **B,** Short-term persistence is shown as CFU from colonized L4 worms that were kept in buffer for 1h, for ancestral and evolved co-cultures. For both measures of host-association, MYb11 and MYb71 abundances within the co-culture are shown for the ancestral (dark magenta/green) and host-evolved (light magenta/green) bacteria are shown on the left. On the right, total colonization levels (i.e., the sum of MYb11 and MYb71 CFUs) are shown for ancestral and evolved bacteria. Replicates are shown as individual data points, and box plots summarize median (central line), upper/lower quartiles (box limits), and the interquartile range (whiskers).
